# A metaverse laboratory setup for interactive atom visualization and manipulation with scanning probe microscopy

**DOI:** 10.1038/s41598-025-01578-y

**Published:** 2025-05-20

**Authors:** Zhuo Diao, Hayato Yamashita, Masayuki Abe

**Affiliations:** Graduate School of Engineering Science, Osaka University, 1-3 Machikaneyama, Toyonaka, Osaka, 560-8531 Japan

**Keywords:** Characterization and analytical techniques, Microscopy, Information technology

## Abstract

We present a metaverse laboratory system that integrates mixed reality (MR) technologies with scanning probe microscopy (SPM) for interactive atomic-scale visualization and manipulation. In order to accommodate both the visualization and input of SPM data in a virtual environment and the physical interaction with SPM-related equipment in the laboratory, the system incorporates a virtual reality (VR) and augmented reality (AR) framework to enable seamless switching between these two environments. Utilizing the pose-tracking capabilities in AR, users can intuitively interact with virtual interface elements and three-dimensional objects through physical hand gesture input to control SPM parameters and probe positioning. The system provides real-time visualization of scanned surfaces at the atomic scale in the virtual environment, enabling immediate feedback during experiments. To demonstrate the system’s capabilities, we performed atomic manipulation experiments using hand gestures for lateral probe positioning, showing how MR-enhanced SPM can simplify nanoscale operations and improve experimental efficiency. Our integrated MR–SPM system allows users to conduct experiments via the metaverse platform while enhancing the human-instrument interaction experience. It extends the practical utility required for both real-time physical and virtual environment SPM operations in the laboratory, making nanoscale research more accessible and intuitive.

## Introduction

Metaverse technologies, such as virtual reality (VR), augmented reality (AR), and mixed reality (MR), have gained significant attention as emerging platforms that transcend physical constraints and accelerate the digitalization of society.^1^ These immersive technologies provide intuitive and interactive interfaces that enhance efficiency and adaptability in business^2^, education^3^, and healthcare^4^. The applications also span multiple scientific domains, including data visualization^5–7^, scientific computing^8,9^, and experimental sciences^10–13^. In laboratory settings, metaverse technologies enable enhanced experimental operations and data exploration, potentially leading to more efficient research workflows. For training purposes, these platforms allow users to practice experimental procedures in a virtual environment before using actual equipment. While the implementation of metaverse technologies in laboratories is still in its early stages, their integration shows promising potential for advancing scientific research methodologies.

One area of scientific research where metaverse technologies can be particularly beneficial is nanoscience and nanotechnology. In these fields, scanning probe microscopy (SPM) is widely used to study nanoscale properties across materials science and biological systems. SPM is a powerful technique for measuring surface structures and properties with resolution ranging from nanoscale to atomic scale. When operating at its highest performance, it enables atomic-resolution imaging, providing information about surface topography as well as electrical, magnetic, and mechanical properties. These capabilities, combined with its ability to operate in various environments including liquids and controlled atmospheres, make SPM an essential tool for investigating both materials and biological systems. Despite its capabilities, SPM operation requires significant expertise, from sample preparation to optimization of measurement parameters and data analysis. Achieving atomic-resolution imaging and spectroscopy demands particularly advanced skills, which can present a substantial challenge for new users.

To address these operational challenges, the integration of metaverse technologies with SPM offers a potential solution. By virtually recreating SPM, it provides a simplified and intuitive operating environment^14,15^, making beginners easier to experience SPM. Furthermore, this integration facilitates remote experiment operation and promotes collaborative research across institutions^9^. The synergy between metaverse and SPM not only enhances education by providing accessible training platforms but also advances surface science research. Simulating and optimizing experimental conditions in metaverse also enables efficient experimental design^7^. Recent advancements in microscopy systems have focused on integrating head-mounted displays (HMDs) with VR computer graphics and implementing hand-controlled manipulation^15–17^ to gain a deeper intuition of scientific phenomena in experimental instruments. Through these developments, we anticipate the emergence of innovative SPM experimental systems that combine virtual and physical capabilities. However, pure VR environments isolate users from the physical laboratory space, potentially limiting their ability to maintain awareness of crucial experimental conditions and safety considerations.

In response to these challenges, the MR technique combining VR with AR offers a more comprehensive solution by overlaying virtual objects onto the real world. The scene understanding capability enables interaction with digital SPM data while maintaining direct visual contact with the physical experimental setup. Additionally, the pose-tracking technique provides a natural hand input interface for engaging with three-dimensional data, thereby improving the efficiency of spatial relationship-based experiments. This physical hand-based interaction method unifies both the physical and virtual inputs within the laboratory environment. After integrating real-space tracking functionalities from AR into SPM, the system creates a more practical and efficient user experience compared to VR-only solutions.

In this study, we have developed an MR–SPM system that introduces gesture-based probe manipulation and imaging control, enabling intuitive operation analogous to robotic arm control. MR seamlessly blends virtual elements with the real laboratory environment, enabling the simultaneous monitoring of both virtual and physical aspects of experiments. The SPM operations represent a significant advancement over traditional SPM control methods, combining the accessibility of MR interfaces with the precision required for atomic-scale operations. Our MR–SPM system complements traditional atomic resolution imaging and manipulation capabilities by providing an enhanced spatial perspective that helps operators better conceptualize three-dimensional atomic arrangements, particularly useful for complex manipulation sequences at room temperature.

## MR–SPM system

### The concept of the metaverse laboratory setup

**Fig. 1:**
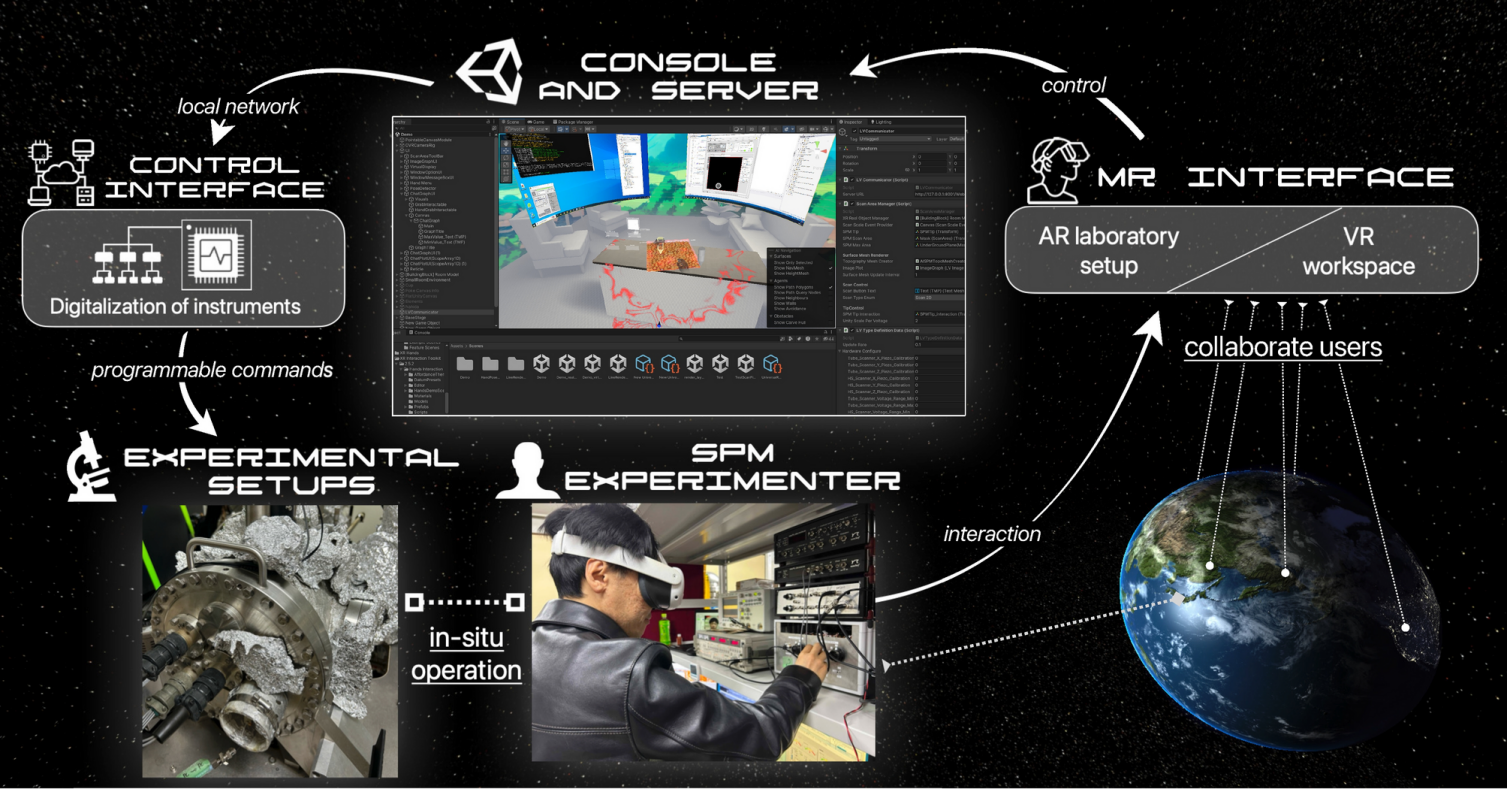
The concept of the mixed reality scanning probe microscopy (MR–SPM) system, including several components for the metaverse laboratory setup.

Figure 1 illustrates our MR–SPM framework, which integrates VR for collaborative experimentation and AR for real-world environment tracking. The system consists of three key components:

1. Control Interface: An FPGA device digitalizes the experimental setup, enabling precise control of instruments via TCP commands over a local network. When designing such a system, it is important to provide customizable APIs that allow user modding to implement additional SPM functionalities^18,19^.
2. Console and Server: Implemented in Unity, this component serves as the VR workspace client and executes user commands to control the instruments. The administrator’s view is shown in Fig. 1, which allows monitoring of the user’s movements (red rim effect and hands rendering) and managing the virtual environment’s parameters and objects through the sidebar menu.
3. MR Interface: An adaptive transition system between VR and physical visuals, a pose tracking input system, and a hand-gesture UI allow users to simultaneously interact with virtual and real environments.

The VR workspace enables researchers worldwide to conduct experiments unconstrained by physical labs^20^, while AR enhances the system with body feedback mechanisms like pose tracking^21^ and simultaneous localization and mapping^22^. Merging VR and AR facilitates seamless data integration between real and virtual spaces. AR-assisted manual interfaces guide users through instrument operation by overlaying visual instructions^23–25^. The MR system accommodates diverse laboratory instruments requiring physical interaction by enabling transitions between VR and traditional visuals, enhancing the experimental workflow and user experience.

### Implementation of MR interface for SPM

**Fig. 2:**
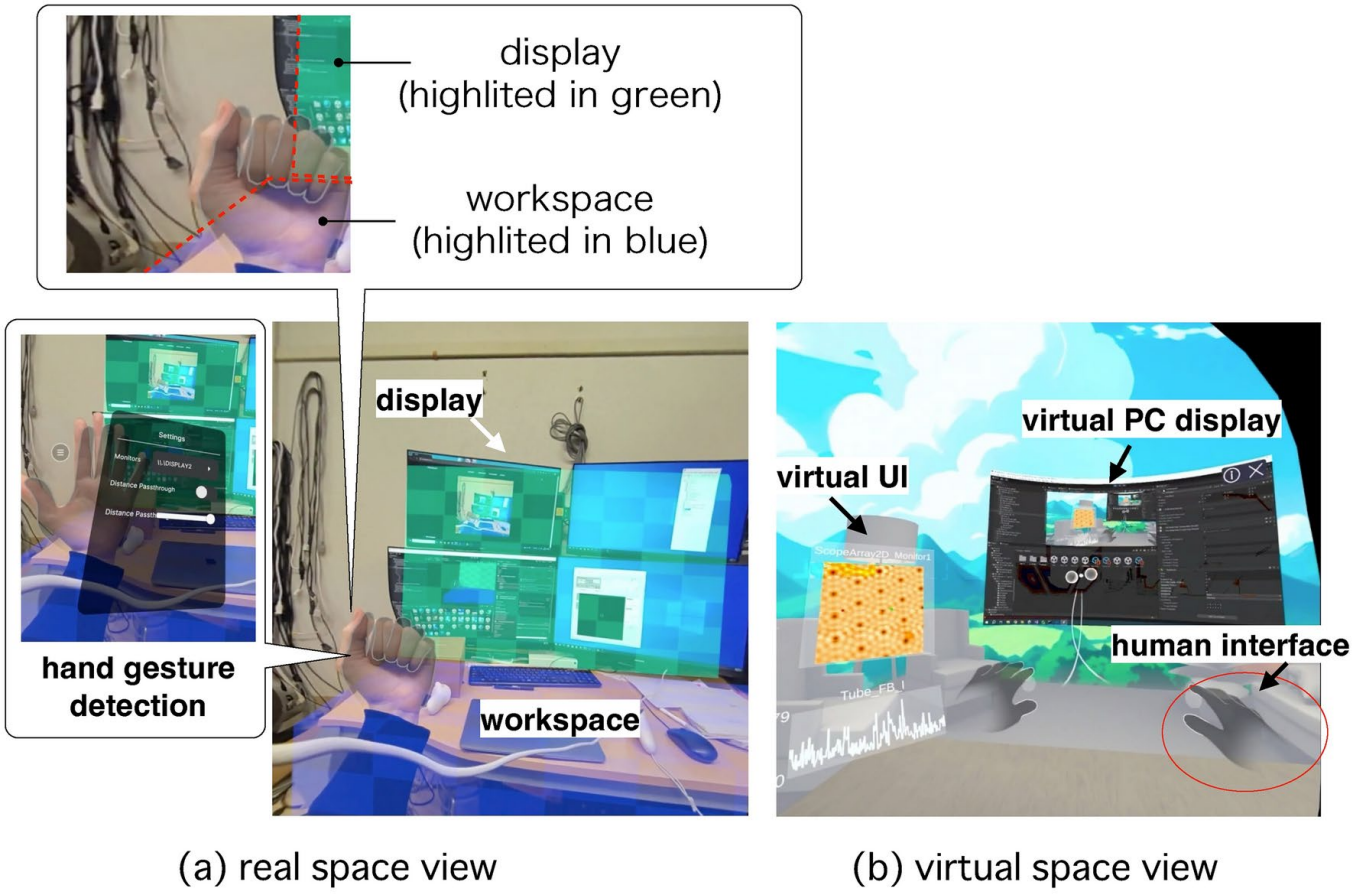
View of the mixed reality scanning probe microscopy (MR–SPM) experiment being conducted through the headset, with automatic switching between (**a**) real space view and (**b**) virtual space view to enable the conventional SPM control on a personal computer. The multimedia file [Movie S1.mp4] illustrates the object tracking of real space.

Figure 2 shows the view seen by the experimental operator while wearing a headset during the MR–SPM experiment. The headset provides the user with automatic switching between (a) a real space view and (b) a virtual space view, enabling conventional SPM control on PC. In the real space view (Fig. 2a), the SPM control interface display is automatically highlighted with a transparent green overlay, while the workspace on the desk is highlighted with a transparent blue overlay, as shown in the magnified callouts above the figure. These regions are pre-registered as marked objects to be detected by the MR system. Hand gesture detection enables interaction with the screen and various menu options in the real-world view, as illustrated in the callout on the left side of the figure.

The virtual space view (Fig. 2b) shows the virtual PC display that the user sees, containing the SPM control software user interface. The virtual UI is aligned with the real workspace using the desk as a reference marker. A virtual user interface has been implemented to monitor scanning signals and provide buttons for operating the equipment. The user can interact with these controls by performing specific hand gestures. Furthermore, by using a virtual PC monitor, standard computer operations can be reproduced even while wearing the headset. Typically, scientific experimental systems require multiple PC monitors to monitor complex operation windows and multi-channel signals. However, in the MR system, these physical monitors can be replaced with corresponding virtual UIs, significantly reducing equipment and space costs. This demonstrates the advanced capabilities of MR–SPM in bridging the physical and digital domains, enhancing user experience and functionality.

### Adaptive switch of virtual and real views

**Fig. 3:**
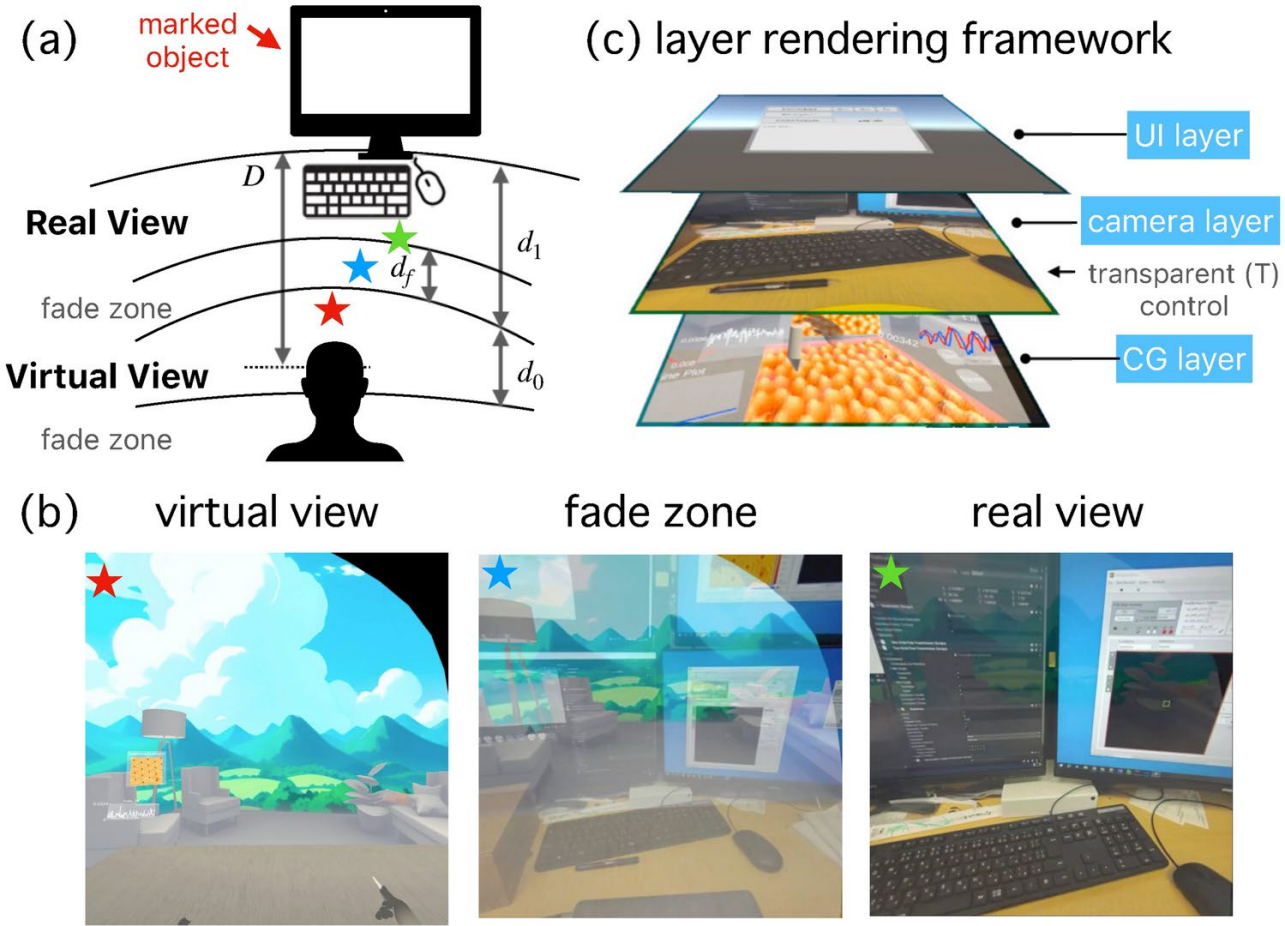
Seamless integration of virtual and real spaces in the mixed reality experimental system. (**a**) Automatic view switching based on the distance between the marked object (desktop PC display at this time) and the user’s face position. A fade area ensures a gradual transition between views. (**b**) Real-time switching between physical and virtual views during an SPM experiment. (**c**) The layer rendering framework implemented for smooth view transitions. The multimedia file [Movie S2.mp4] illustrates the transition between real and virtual views.

In our mixed reality system, precise probe positioning and surface imaging can be performed in the virtual reality (VR) environment. However, VR alone does not allow for direct interaction with physical instruments, which is crucial during the experiment. To overcome this limitation and enable the operator to control multiple instruments simultaneously, a real-world view must be incorporated. Seamlessly switching between the virtual and physical workspaces is essential for maintaining the efficiency and accuracy of the experiment. To address this requirement, we have developed a framework that balances both virtual and real-world views, as depicted in Fig. 3. Our system allows for smooth transitions between VR and the real-world view, ensuring that the operator can effectively interact with the physical instruments while benefiting from the precision and visualization capabilities provided by the VR environment.

The virtual and real views are designed to switch dynamically based on the current task requirements, which can be detected from the relative world position of the user’s head anchor and the marked object in the real world. Figure 3a illustrates an example where we set the PC display as the “marked” object. When the experimenter is near the marked object, the transition between the real and virtual views is achieved through a fade zone, defined by the distances *D* from the marked object. When the user is located beyond $d_1$, the system displays a fully virtual view. As the user moves closer to the marked object and enters the fade zone $d_f$, the system gradually changes the contrast from real to virtual. This transition is completed when the user reaches $d_1$, and the view becomes fully real. The virtual view persists until the user moves beyond $D=d_1+d_0$, at which point the system switches back to the real view. This approach ensures a smooth transition between the real and virtual environments, enhancing the user’s immersion in the experimental experience while minimizing distractions from real-world elements. The transparency *T* dynamically changes in real-time depending on the distance *D* between the user’s head anchor and the target real object, according to the following equation:

1 $$\begin{aligned} T = {\left\{ \begin{array}{ll} 1 & (0 \le D \le d_1 - d_f) \\ \frac{d_1 - D}{d_f} & (d_1 - d_f \le D < d_1) \\ 0 & (d_1 \le D \le d_1 + d_0) \\ 1 & (d_1 + d_0 \le D) \end{array}\right. } \end{aligned}$$

As the user approaches the target, the system is designed to fade the view to the real world gradually. This transition eventually reaches a state where the user can interact with the real object while still maintaining the visibility of relevant virtual elements [Fig. 3b]. To correspond with the real view, the VR scene background consists of six high-resolution (8192$\times$8192) textures with distinct scenery for each direction, arranged in a hexagonal tiling. By aligning the real and VR backgrounds, users can smoothly transition between the two environments, preventing abrupt changes that might cause disorientation and ensuring a seamless and intuitive experience. During the gradual transition, users can maintain their sense of direction by corresponding to the real-world and VR backgrounds, which helps prevent abrupt changes that might cause disorientation, ensuring a smooth and intuitive experience. To achieve this implementation, we implemented a 3-layer rendering pipeline, as illustrated in Fig. 3c. This pipeline consists of a CG layer, a camera layer, and a UI layer. These layers are rendered sequentially to produce the final composite image displayed on the VR screen. When $T = 1,$ the camera layer completely overwrites the CG layer, causing the game engine to display real-world imagery. Conversely, when $T = 0$, the camera layer is fully transparent, allowing the virtual CG layer to be fully visible. For values of *T* between 0 and 1, a blend of virtual and real imagery is presented, with the proportion determined by the value of *T*.

### Hand gestures

Hand gestures play a fundamental role in handling SPM experiments operation, serving as an alternative to hand-held VR controllers. For MR systems designed for scientific experiments, hand gesture control provides a unified interaction method that enables seamless manipulation of both physical experimental instruments and virtual objects. This interface offers an alternative approach to operating SPM probes that leverages natural hand gestures and spatial awareness. The immersive 3D environment makes atomic manipulation more intuitive for novice operators and provides experienced users with clearer contextual information during complex tasks. Hand gestures are particularly beneficial for precise tasks in SPM experiments, such as atom manipulation, as they can replace traditional input devices like keyboards and mice. This allows users to input tree-dimensional spatial data, including coordinates and trajectories, more intuitively.

**Fig. 4:**
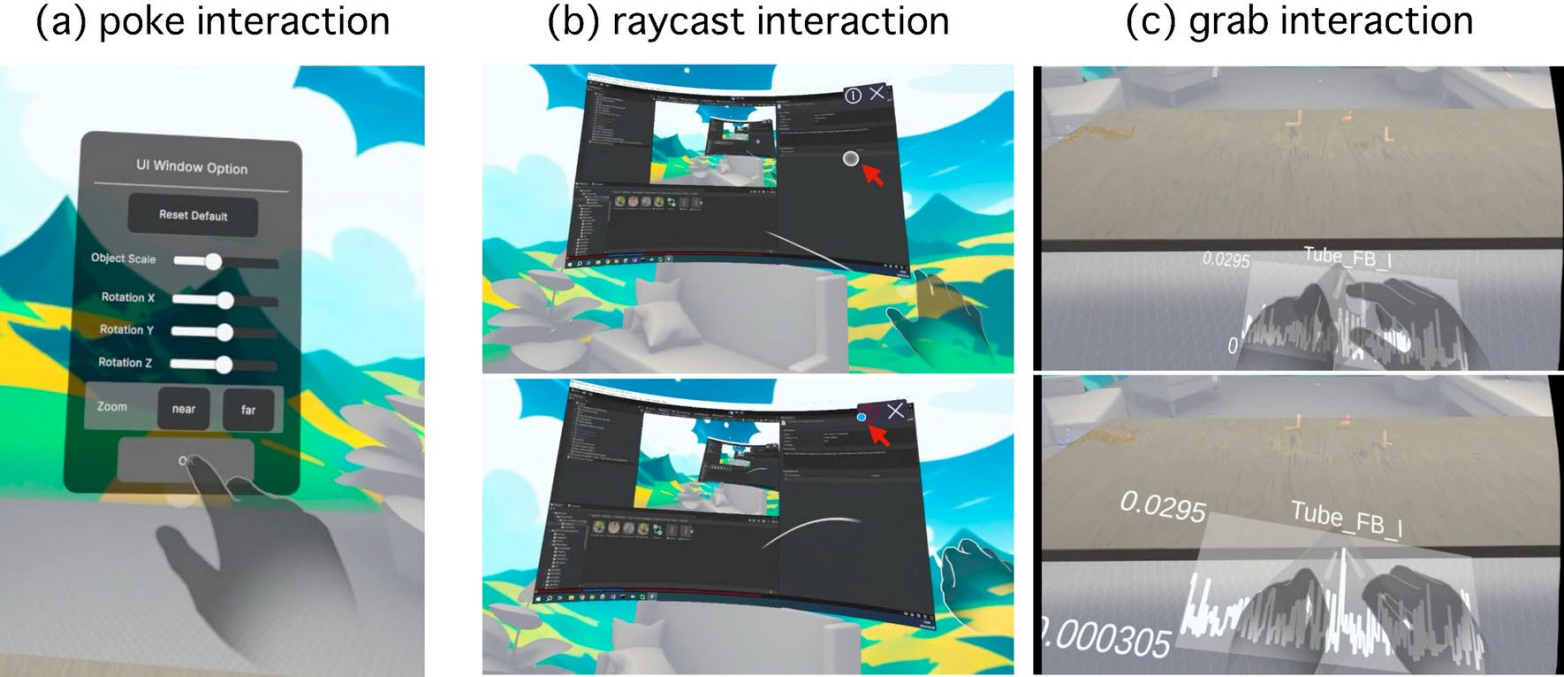
Hand gesture interface to interact with virtual UI and objects. The multimedia files [Movie S3a.mp4, Movie S3b.mp4, and Movie S3c.mp4] respectively illustrate the usage scenarios of the three interactive systems.

Figure 4 illustrates three primary hand gesture interaction methods for interacting with virtual user interfaces (UI) and objects in a mixed reality (MR) environment. (a) depicts the poke interaction, where the user directly touches a UI panel on a virtual window to configure options. (b) shows the raycast interaction, which allows users to operate virtual objects from a distance by aiming at a floating virtual display. (c) demonstrates the grab interaction, enabling users to directly grasp and manipulate virtual objects that display data graphs and numerical values, providing a more tangible and intuitive interaction experience. The grab interaction allows users to adjust object transformations such as scale, rotation, and position using hand gestures. These three interaction methods provide users with intuitive and effective ways to interact with virtual objects and information in an MR environment. Each technique is suited for different situations and levels of precision, offering a versatile interaction framework that can adapt to various user requirements and scenarios.

## Experimental results

### SPM imaging operations in VR workspace

**Fig. 5:**
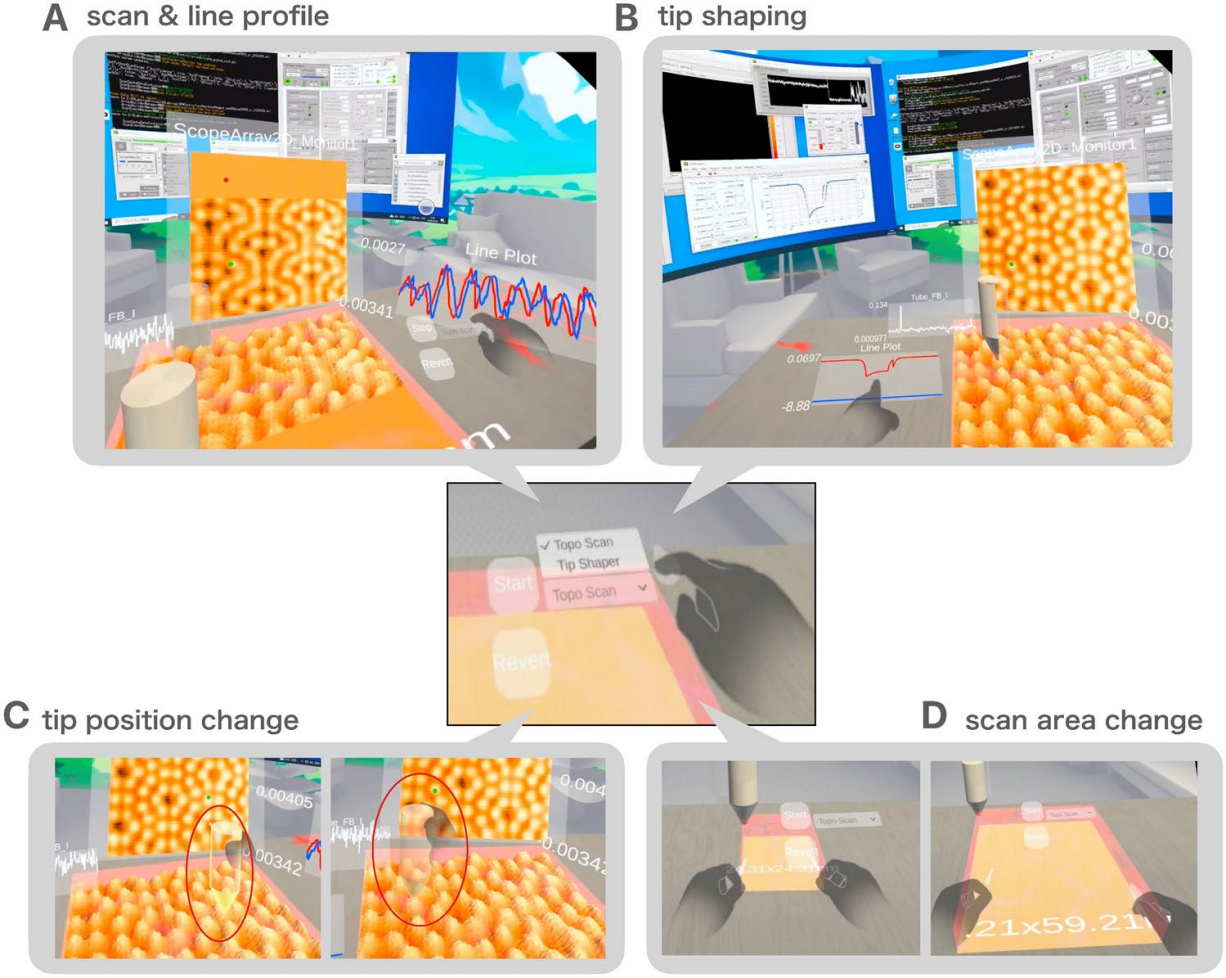
Rendering in the SPM imaging in the VR workspace. The users can use gestures to display windows to be displayed in the virtual space. Here, as an example, the normal scanning screen, line profile display, tip shaping, moving the probe, and changing the scanning range are shown. The multimedia files [Movie S4a.mp4] and [Movie S4b.mp4] respectively illustrate the topographic scan scene and the atomic scan scene. The multimedia file [Movie S4c.mp4] demonstrates the adjustment of the scan range using hand gestures.

Figure 5 illustrates performances of typical SPM operations performed in the VR workspace. These results are obtained as the user interacts with the virtual environment using gestures while conducting experiments. The operations can be selected from the menu using hand gestures, as shown in the central panel of the figure. Panel A showcases the ’scan & line profile’ feature. In normal imaging mode, it is possible to acquire images in real-time and display them in both 2D and 3D. Additionally, line profiles can also be displayed to provide cross-sectional information about the surface topography. In the B panel, the ’tip shaping’ functionality is showcased, enabling researchers to modify and optimize the probe tip for improved experimental performance by adjusting the distance between the probe and the sample and the applied voltage^26^. The C demonstrates the “tip position change” capability, where users can precisely adjust the probe’s location on the sample surface. The D panel illustrates the ‘scan area change’ function, which enables users to dynamically alter the scanning range to focus on specific regions of interest, allowing for a flexible selection of areas for detailed analysis.

### Mechanical vertical manipulation of selected single atoms

In addition to atomic-scale imaging, we demonstrated mechanical vertical atomic manipulation^27^ in VR space using this system at room temperature. Atomic manipulation technology represents a significant advancement in the field of nanoscience. This technique enables precise control and manipulation of individual atoms at the atomic scale^28^, dramatically deepening our understanding of material structures and properties at the atomic level. By controlling the arrangement of atoms and molecules at will, various applications are anticipated, including not only the construction of novel atomic structures but also the fabrication of atomic-scale logic circuits^29^ and memory devices^30^, and the control of chemical reactions at the single-atom level^31,32^ . MR–SPM has the potential to become a powerful platform that enables real-time visualization of these atomic manipulation processes and allows for intuitive operation.

**Fig. 6:**
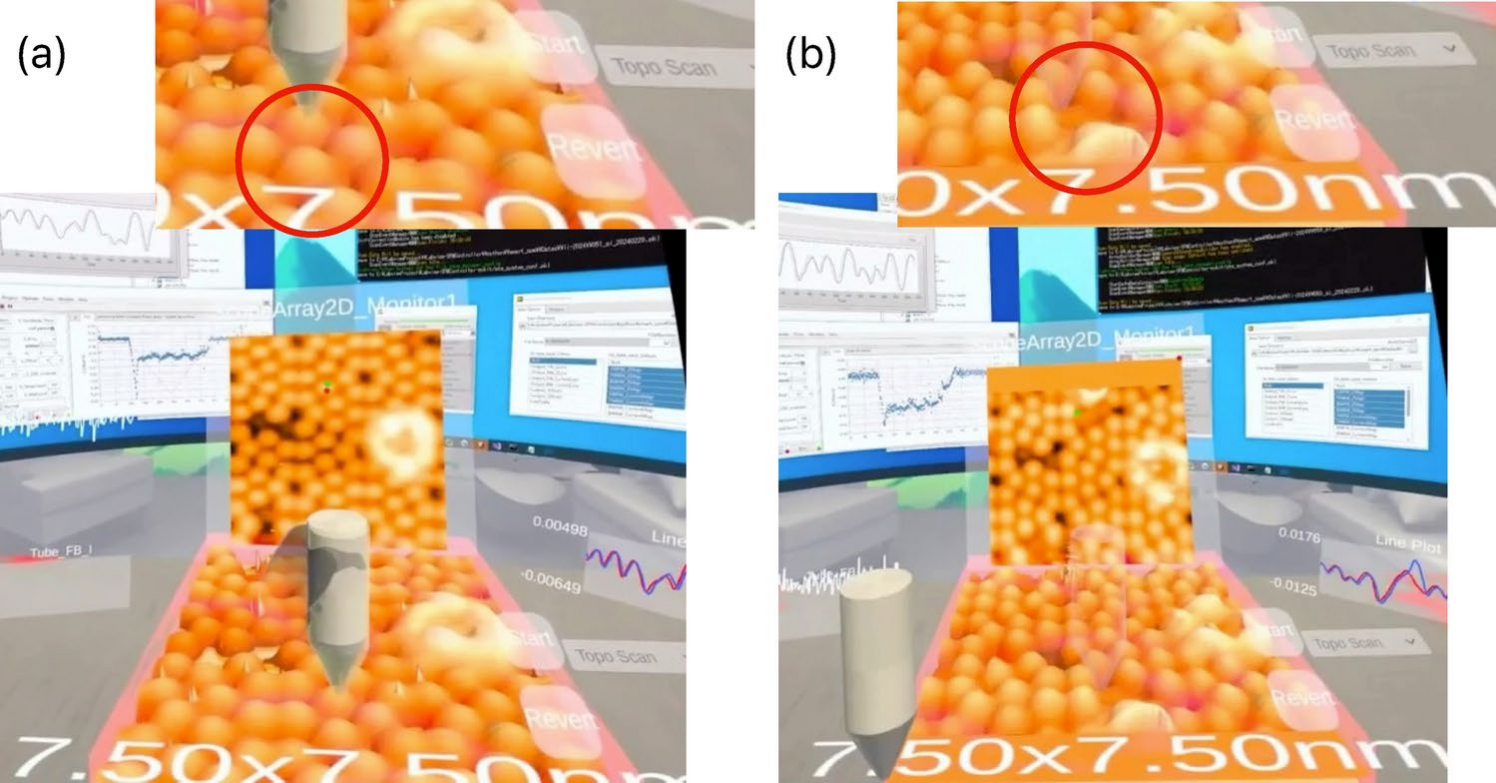
Mechanical vertical atom manipulation using MR–SPM at room temperature. This experiment demonstrates the selective extraction of individual silicon atoms from a Si(111)-(7×7) surface. (**a**) and (**b**) show the VR workplace views from different perspectives before and after the atomic extraction process, respectively. The STM probe was positioned over the targeted Si atom to be manipulated using lateral (X-Y) gesture controls in the VR environment.

As shown in Fig. 6, we conducted vertical atomic manipulation experiments in the VR environment. The STM probe was moved using hand gestures in VR space to extract atoms mechanically. In this experiment, we utilized the gesture functionality of the VR system to manually position the STM tip above the target silicon atom on the sample surface. In this experiment, the operator’s tip movement was restricted to directions parallel to the sample surface. Since vertical atomic manipulation requires precise control of the tip-sample distance, we decided not to use gestures for vertical tip control to prevent unintended tip-sample crash interactions. Figure 6a,b show STM images before and after the experiment as displayed in VR space, depicting the same 7.50 $\times$ 7.50 nm scan area from different perspectives. The red circles in both images indicate the location of the extracted silicon atom. The experimental results demonstrate successful selective extraction of the targeted silicon atom in VR space, with the change clearly visible in the STM images. Notably, the post-extraction STM image revealed a characteristic vacancy where the target atom had been removed, while maintaining the surrounding atomic structure intact.

## Discussion

The atomic manipulation experiments demonstrated that VR-based gesture control enables more intuitive and rapid probe positioning compared to conventional interfaces. While traditional two-dimensional displays certainly provide continuous feedback, three-dimensional visualization offers complementary spatial context that can be particularly valuable when planning and executing multi-step atomic manipulations that require an understanding of complex three-dimensional relationships. Future improvements should focus on system stability and precision through automated control functions. Machine learning for optimal probe path calculation and haptic feedback implementation could enhance atomic-level manipulation capabilities. Currently, probe movement is limited to X and Y directions to prevent equipment damage. Incorporating Z-axis control with an integrated haptic feedback system^33,34^ could provide real-time tactile perception of probe-surface interactions, enabling safer three-dimensional manipulation. A key technical limitation is scanning speed, with our system requiring 30 seconds per area scan. To match human visual perception, the surface structure rendering should update within 15 ms^35^. Recent advances in hardware^36^ and software^37^ implementations have demonstrated video-rate scanning capabilities. In the system architecture of the SPM and MR application client, multi-processing needs to be implemented to reduce CPU load and increase the update rate for data handling. Achieving instantaneous surface rendering would significantly enhance atomic manipulation, spectroscopy measurements, and other precise positioning applications.

The MR–SPM system demonstrates the potential integration of metaverse technology with scientific instrumentation. The MR system utilizes a hand gesture interface, allowing users to intuitively understand the instrument’s operation through complex workflows. The scanning results are rendered by computer graphics, making atomic-scale phenomena more vivid and tangible. This advancement not only makes the system more accessible for education but also enables remote experimentation and efficient research collaboration. Further optimization of user interaction can be achieved by introducing a language model-driven SPM system^38–40^ to simplify device control and by integrating autonomous experimentation algorithms^19,41^ to replace manual operations in executing specific experimental workflows. By implementing these capabilities, future metaverse laboratory platforms could enable users to focus on analyzing data rendered more intuitively within a 3D virtual space, while the experiment could be solely conducted via general decision-making through direct interaction, such as hand gestures. Our system completes the SPM instrument digitalization, allowing researchers to conduct experiments from any location with minimal on-site administration. This system makes multi-user participation in experiments on the MR platform possible, thus manifesting an SPM metaverse laboratory.

Despite the promising capabilities of our mixed reality interface for SPM, several important challenges and limitations should be acknowledged before deploying as a public application. The laboratory’s real-world mapping relies on pre-calibration, which limits its ability to detect dynamic objects in laboratory environments. This makes MR headset users less environmentally aware and creates potential safety hazards during experiments. Mapping algorithms suited for indoor scenes^42^ with lower computational costs may be introduced to ensure a safer user experience. Current commercial hardware faces significant limitations, such as excessive weight ($\approx$500g) and short battery life ($\approx$2 hours), which contribute to user fatigue during extended sessions. Although VR displays typically project images at a distance of around 2 m, potentially reducing certain types of focus strain for eyes compared to conventional monitors, MR systems with binocular displays still suffer from the persistent vergence-accommodation conflict^43^, which exacerbates visual fatigue and limits comfortable headset use. Addressing these ergonomic challenges is essential for enhancing long-term usability and user adoption. Additionally, in a cross-border metaverse laboratory, user regulation and security concerns may raise a problem^20^. In MR applications, user data streams are presented in more diverse ways, making real-time communication management more challenging compared to conventional social media platforms. Addressing these limitations can expand the applicability of metaverse laboratories.

In conclusion, we developed an MR interface that seamlessly integrates real and virtual laboratory environments for SPM experiments. While wearing an HMD, operators can efficiently transition between physical equipment control and virtual system interaction. The system’s tree-dimensional visualization enhances spatial awareness and enables intuitive configuration of measurement parameters based on tip-sample interactions. We demonstrated the system’s capabilities through successful atomic manipulation experiments on Si(111)-(7×7) using hand gesture controls. This integration of SPM with metaverse technology represents a significant advancement in materials science experimentation.

## Methods

### MR–SPM system

The experiments were conducted using a home-built SPM operated at room temperature in an ultra-high vacuum environment ($< 1 \times 10^{-8}$ Pa). An Si(111)-(7×7) surface was prepared using the standard flashing-annealing method. STM images were acquired using a Pt/Ir probe. To integrate SPM instrument and MR, a custom system was developed using LabVIEW and Python for SPM data acquisition. The measurement board employed was a PXIe-7857R from National Instruments. The MR headset used was a Meta Quest 3 from Meta. The MR component of the program was developed via the Unity platform, while the SPM remote program was implemented in Python.

### Mixed-reality environment setup

The Meta Quest 3 serves as the head-mounted display (HMD), while Unity functions as the computer graphics renderer and game engine to host the MR system. This setup runs on a PC equipped with an Intel i9-9900K CPU and NVIDIA RTX 4090 GPU. Data communication between the Meta Quest 3 and Unity is facilitated via Oculus Link. To implement MR features in Unity, we utilize Meta XR SDKs. Within the Unity component of the Meta XR SDKs, the behavior of virtual UI and virtual game objects is constructed through Unity components based on Hand Poke Interactive, Ray Interactive, and Hand Grab Interactive components. Additionally, tip interaction is facilitated by the Hand Pose Detection component. The spatial data of real objects is referenced from the Meta Quest 3 scene, which can be preliminarily set up to include object labels and detected surfaces. In Unity, the “HTTP GET” method is employed to acquire application cache data via the HTTP server module on the SPM application, while the “HTTP POST” method is utilized for data transfer and remote scanning. Given that we run the HTTP server on localhost, the latency between the MR environment and the SPM system is less than 10 ms. Considering the latency in the instrument data processing framework and the load on the CPU, the update rate of the thread monitoring data communication in the MR interface is set to 60 Hz, while the update rate for the thread acquiring and updating measurement data in the SPM system is set to 20 Hz.

### Atom manipulation method

This research involves the manipulation of a Si dopant atom on a Si(111)-(7×7) surface using the “atomic pen technique”^44^ at room temperature. Initially, we perform an automatic drift compensation^45^ method to ensure that the distance between SPM probe and sample can be located precisely. Next, the system maintains the Z feedback and positions the SPM probe precisely over the target atom’s X and Y coordinates. Then, the atomic scan is activated, periodically pausing the Z feedback and gradually lowering the probe to a predetermined distance from the surface. This process continues until a specific and significant change is observed in the atomic scan signal which can indicate the successful removal of the target Si adatom.

## Supplementary Information

Supplementary Information.

Supplementary Movie S1.

Supplementary Movie S2.

Supplementary Movie S3a.

Supplementary Movie S3b.

Supplementary Movie S3c.

Supplementary Movie S4a.

Supplementary ViMoviedeo S4b.

Supplementary ViMoviedeo S4c.

## Data Availability

The datasets used and/or analysed during the current study are available from the corresponding author on reasonable request.
