# Supplementary material for: A metaverse laboratory setup for interactive atom visualization and manipulation with scanning probe microscopy
Source: Sci Rep. 2025 May 20;15:17490. doi: 10.1038/s41598-025-01578-y (PMC12092676; doi:10.1038/s41598-025-01578-y)
Supplement: Supplementary file 1 — Supplementary Information. [file 41598_2025_1578_MOESM1_ESM.pdf]

Supplementary of A metaverse laboratory setup for interactive atom visualization and manipulation with scanning probe microscopy

Zhuo Diao[1,*], Hayato Yamashita[1], Masayuki Abe[1,+]

[1]Graduate School of Engineering Science, Osaka University, 1-3 Machikaneyama, Toyonaka, Osaka 560-8531, Japan.
*diao.zhuo.es@osaka-u.ac.jp.
+abe.masayuki.es@osaka-u.ac.jp.

Contents

1 Movie S1 caption. 1

2 Movie S2 caption. 1

3 Movie S3 caption. 1

4 Movie S4 caption. 2

1 Movie S1 caption.

This video demonstrates the object tracking capability in real space, as illustrated in Figure 2 of the manuscript. In our laboratory, the system recognizes and highlights the display in the green region and the workspace in the blue region. Additionally, it tracks user hand gestures and displays a menu when the palm is open.

2 Movie S2 caption.

This video demonstrates a seamless switch of virtual and real views in the mixed reality experimental system, as illustrated in Figure 3 of the manuscript. Our system is designed to immerse users in virtual reality while allowing interaction with real-world objects. When the user approaches a designated real-world object (in this case,

the display), the system automatically switches to a real-world view, enabling the user to observe and manipulate the physical object.

3 Movie S3 caption.

This video demonstrates three hand gesture interfaces for interacting with virtual UI and objects, as illustrated in Figure 4 of the manuscript.

- Movie S3a.mp4 allows users to interact with the UI by touching a virtual menu.
- Movie S3b.mp4 enables users to perform mouse-like click operations by targeting distant UI elements.
- Movie S3c.mp4 allows users to directly manipulate virtual 3D objects, enabling changes to their position, rotation, and scale.

4 Movie S4 caption.

This video demonstrates the implementation of the MR-SPM workspace, as illustrated in Figure 5 of the manuscript.

- Movie S4a.mp4
  It shows the control and observation of an SPM topography scan within the VR interface. The system acquires topography data from the SPM system during scanning and renders it in the "ScopeArray2D_Monitor" window, performing real-time surface 3D modeling. Additionally, it visualizes the obtained line profile with forward and backward direction signals. The "Tube_FB_I" window displays the real-time tunneling current signal in nA. The signal value remains around 0.03 nA because the scan setpoint is 30 pA.
- Movie S4b.mp4
  This illustrates an atomic scan performed by interacting with a virtual SPM tip. The normal tip represents the current position of the probe, while the ghost-like tip serves as an interface for controlling its movement. The ghost-like tip can be moved using a grab gesture. When a hand is placed on it, it will hover in yellow if it is in a controllable state. After positioning the probe at the desired scanning location based on the 3D surface model, a 1D scan can be performed at the corresponding position using the atomic scan option in the menu. The ghost-like tip's position serves as a reference for the last atomic scan location.
- Movie S4c.mp4
  This demonstrates the ability to expand, shrink, and move the scan area through the manipulation of virtual 3D objects shown in Movie S3c.mp4. In the video, the text below the scan area displays the current scan range size, which is updated in real time through interaction with the SPM system.
